# Niche comparison among two invasive leafminer species and their parasitoid *Opius biroi*: implications for competitive displacement

**DOI:** 10.1038/s41598-017-04562-3

**Published:** 2017-06-26

**Authors:** Zhenlong Xing, Linya Zhang, Shengyong Wu, Hao Yi, Yulin Gao, Zhongren Lei

**Affiliations:** 1 State Key Laboratory for Biology of Plant Diseases and Insect Pests, Institute of Plant Protection, Chinese Academy of Agricultural Sciences, Beijing, 100193 China; 2 Fujian-Taiwan Joint Center for Ecological Control of Crop Pests, Fuzhou, 350002 China

## Abstract

Fundamental to competitive displacement in biological invasion is that exotic species occupy the ecological niches of native species in novel environments. Contrasting outcomes of competitive displacement have occurred between *Liriomyza trifolii* and *L. sativae* in different geographical regions following their introduction. Various factors have been advanced in an attempt to explain these different competitive outcomes, although none of these explanations have addressed the effects of niche differences. We conducted field cage experiments to compare the feeding and habitat niches of the two leafminer species and their primary parasitoid, *Opius biroi*, when occurring together on kidney bean. A wider spatiotemporal niche breadth was found in *L. trifolii* (0.3670) than in *L. sativae* (0.3496). With respect to the parasitoid, the proportional niche similarity between *L. sativae* and the parasitoid was 0.3936 but only 0.0835 for *L. trifolii*, while similar results were found for niche overlap, indicating that stronger trailing behaviour and parasitic effects of *O. biroi* occurred in *L. sativae*. In conclusion, *L. trifolii* has outperformed *L. sativae* in occupying the ecological niche and is superior to *L. sativae* in avoiding parasitization by the pupal parasitoid, *O. biroi*.

## Introduction

The concept of an ecological niche is fundamental in explaining species interactions and coexistence^1^. Gause contends that two species with similar ecology cannot coexist together in the same location^2^, and when or if these similar species encounter each other or become sympatric, the process of niche differentiation or competitive displacement will occur^3, 4^. Competitive displacement is always associated with biological invasion^5^. Moreover, fundamental to the establishment of non-native species is the requirement that they find an empty ecological niche or occupy the niche of a native species in the new environment^6^. While studying interspecific competition, Denno found that interspecific competition occurred in 76% of 193 pairwise comparisons of phytophagous insect species, particularly when they were closely related or recently introduced^7^. Interestingly, while 84% of the competitive interactions were asymmetric, large-scale competitive exclusion occurred in only 11% and 3% of haustellate and mandibulate herbivores, respectively^7^.

The two agromyzid leaf-mining species *Liriomyza sativae* Blanchard and *L. trifolii* (Burgess) (Diptera: Agromyzidae) are major threats to the production of many varieties of horticultural plants and vegetable crops. The two species, both of which are native to the Americas, have successfully become established in most parts of the world^8, 9^. *L. sativae* and *L. trifolii* were first found in mainland China in 1994 and 2005, respectively^10, 11^. The former has been a prevalent leafminer throughout the mainland following its invasion^12^. Similarly, the latter has now also become widespread in southeast China^13^. Serious interspecific competition has occurred between the two species in areas where they co-occur. In western North America and China, *L. trifolii* has displaced the dominant *L. sativae*, while the opposite has occurred in Japan^14^. These different competitive outcomes have resulted in the publication of several studies involving statistical comparisons of the various traits of the two leafminers. In western North America, a lower susceptibility to insecticides in *L. trifolii* and the utilization of a different host plant were considered to be the cause for this displacement^15, 16^. Similarly, in Hainan province, China, lower insecticide susceptibility coupled with the monocultural planting of cowpeas were considered to be the major factors responsible for the rapid displacement^17, 18^, while in Japan, the displacement has been attributed to the higher fecundity of *L. sativae* coupled with different effects of parasitoids^19^. However, niche effects, which are fundamental to interspecific competition^1^, were not considered in any of the reports explaining the displacement of the two leafminers.

Natural enemies, which are often thought to be a major factor in maintaining pests at low levels, can potentially reduce interspecific competition^20^. However, they may also be a significant factor in mediating interspecific interactions^21^. By experimentally removing the predators of two aphid species, increases in the density of the two species and the competitive interactions between them occurred^22^. A similar phenomenon occurred when parasitoids of hemlock scale insects were excluded^23^. According to Denno’s assessment, approximately 17% of the interspecific interactions of herbivores were mediated by natural enemies^7^. In addition to influencing the intensity of interspecific competition, natural enemies can also alter the outcome of competition between two species and, depending on whether they promote coexistence or accelerate exclusion, will determine which of the herbivores suffers the most predation^7^. The effects of parasitoids on the competitive displacement that has occurred between the two agromyzid leafminers are still not thoroughly understood, although one braconid parasitoid, *Dacnusa sibirica*, has been reported to be more likely to control *L. trifolii* ^24^.

The definition of a niche is complex; it includes the spatial niche, functional niche and n-dimensional hypervolume niche, etc.^25^. In this study, we conducted field cage experiments in an attempt to explore the niche (spatial niche throughout a year) differences between *L. sativae* and *L. trifolii* and to evaluate the asymmetrical effects of a pupal parasitoid (*Opius biroi* Fischer, which is an indigenous and prevalent parasitoid of leafminers in China)^26^ on the two competitive, closely related and highly invasive leafminer species when they occurred together on kidney bean.

## Results

### Population dynamics and spatial distributions of *L. trifolii*, *L. sativae* and *O. biroi*

**Figure 1:**
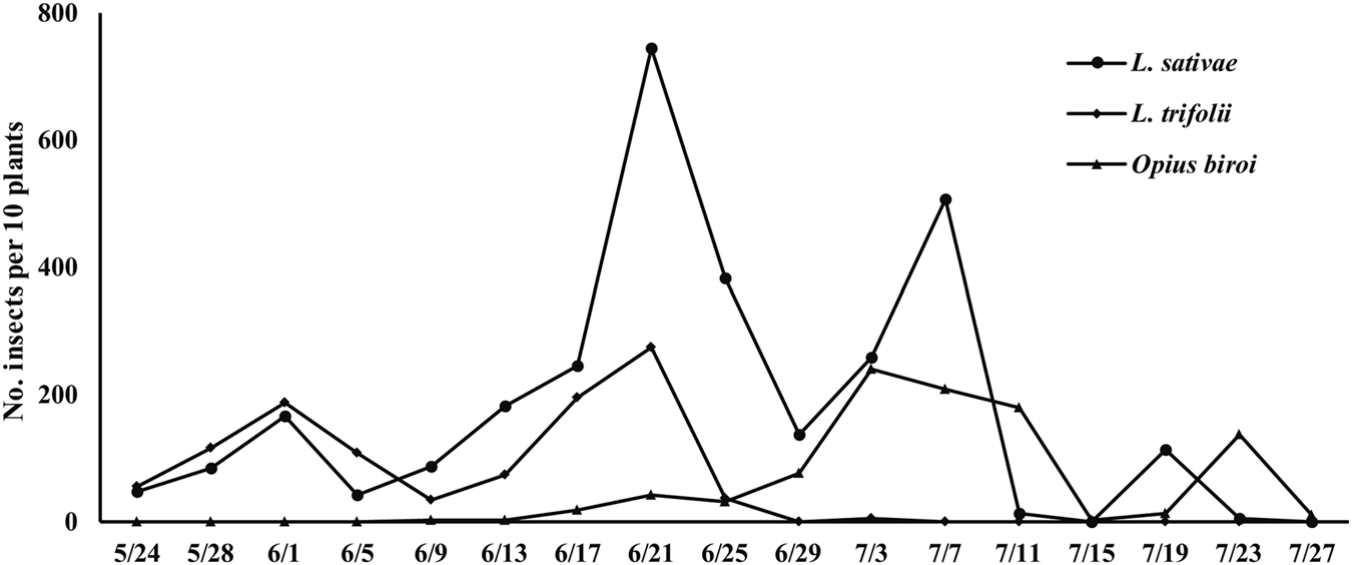
Population dynamics of the two leafminers, *Liriomyza sativae* and *L. trifolii* and the pupal parasitoid, *Opius biroi*.

The populations dynamics of *L. trifolii* and *L. sativae* revealed similar tendencies from May to June. However, densities of *L. trifolii* were higher than those of *L. sativae* from 24 May to 5 June, 2013. Populations of *O. biroi* emerged on 5 June, and their densities first rose and then fell (Fig. 1).

**Figure 2:**
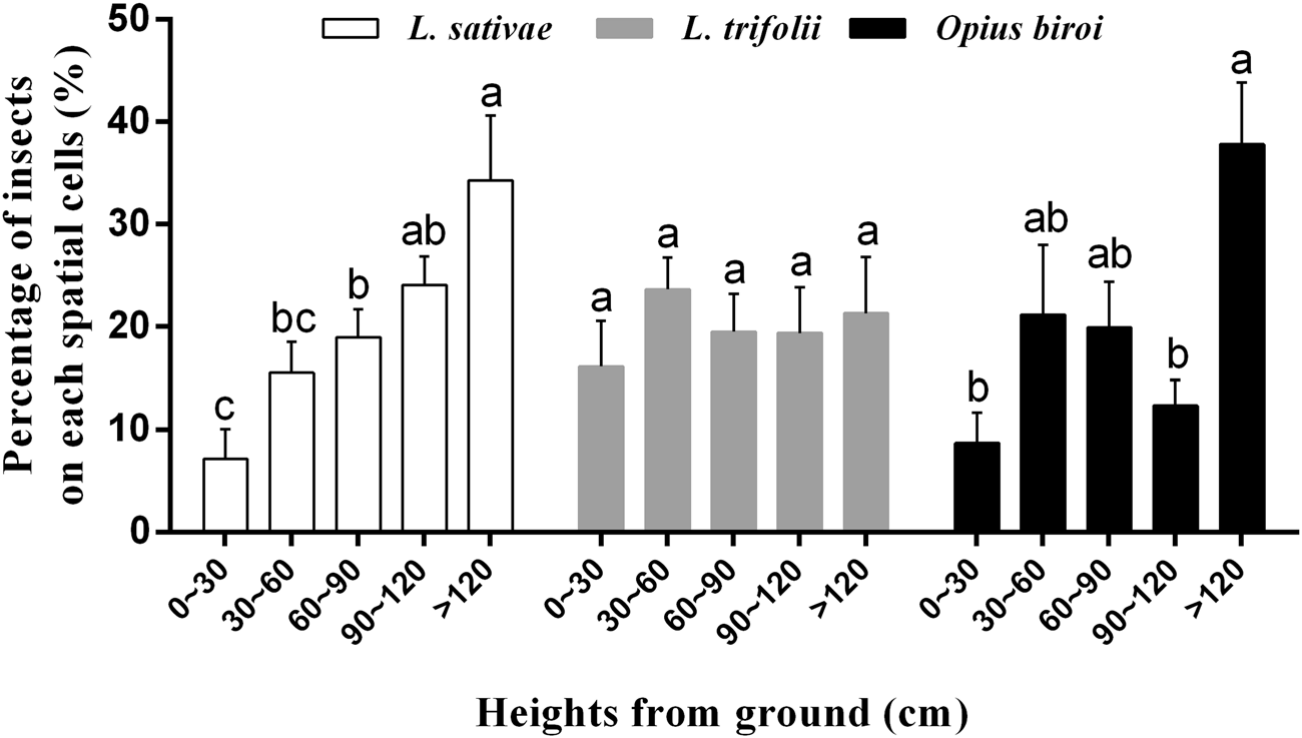
Distribution of *Liriomyza sativae, L. trifolii* and *Opius biroi* in each spatial cell (bars sharing the same letter indicate no significant differences were found at the 5% level using Turkey’s HSD tests).

On the caged kidney bean plants, nearly 60% of *L. sativae* occurred at 90 cm or higher above the ground level (F_4,70_ = 6.14, P < 0.0001), whereas *L. trifolii* were uniformly distributed in each spatial cell (F_4,45_ = 0.41, P = 0.80). The spatial distributions of *O. biroi* were similar to those of *L. sativae* (Fig. 2).

### Niche analysis of *L. trifolii*, *L. sativae* and *O. biroi*

We performed niche analysis based on the population surveying results for the two leafminer species and the braconid pupal parasitoid, *O. biroi*, on caged kidney beans.

Our results showed that *L. trifolii* had a wider two-dimensional niche breadth (0.3670) than *L. sativae* (0.3496) and *O. biroi* (0.2667) (Table 1). The spatial niche of *L. trifolii* (0.9648) was also wider than that of *L. sativae* (0.7783) and *O. biroi* (0.7762) (Table 2). However, *L. sativae* had a wider temporal niche than *L. trifolii* and *O.biroi* (0.4491, 0.3804 and 0.3435, respectively) (Table 3). Each of their temporal niche breadths was below 0.5, whereas their spatial niche breadths were approximately 0.8 (Tables 2 and 3). The spatiotemporal niche overlap between *L. sativae* and *L. trifolii* was approximately 0.6, while the proportional similarity in the spatiotemporal niche was ca. 0.4 (Table 1).

**Table 1:** Spatiotemporal niche of the two leafminer species, *Liriomyza sativae* (LS) and *L. trifolii* (LT) and the pupal parasitoid, *Opius biroi* (OB).

Spatiotemporal niche	Niche breadth			Niche overlap			Niche proportional similarity		
	LS	LT	OB	LS	LT	OB	LS	LT	OB
LS	0.3496			1	0.5730	0.5982	1	0.4048	0.3936
LT		0.3670		0.6015	1	0.1057	0.4048	1	0.0835
OB			0.2667	0.4563	0.0768	1	0.3936	0.0835	1

**Table 2:** Spatial niche of the two leafminer species, *Liriomyza sativae* (LS) and *L. trifolii* (LT) and the pupal parasitoid, *Opius biroi* (OB).

Spatial niche	Niche breadth			Niche overlap			Niche proportional similarity		
	LS	LT	OB	LS	LT	OB	LS	LT	OB
LS	0.7783			1	0.7443	0.9886	1	0.7066	0.9287
LT		0.9648		0.9226	1	0.9385	0.7066	1	0.7512
OB			0.7762	0.9859	0.7550	1	0.9287	0.7512	1

**Table 3:** Temporal niche of the two leafminer species, *Liriomyza sativae* (LS) and *L. trifolii* (LT) and the pupal parasitoid, *Opius biroi* (OB).

Temporal niche	Niche breadth			Niche overlap			Niche proportional similarity		
	LS	LT	OB	LS	LT	OB	LS	LT	OB
LS	0.4491			1	0.7699	0.6051	1	0.5729	0.4238
LT		0.3804		0.6520	1	0.1126	0.5729	1	0.1112
OB			0.3435	0.4628	0.1017	1	0.4238	0.1112	1

### Niche comparison between the two leafminer species and their parasitoid

The niche overlaps between *L. sativae* and *O. biroi* were obviously higher than that between *L. trifolii* and *O. biroi* (the temporal overlaps were 0.6051 and 0.1126, respectively, the spatial overlaps were 0.9886 and 0.9385, respectively, and the spatiotemporal overlaps were 0.5982 and 0.1057, respectively) (Tables 1–3).

The spatiotemporal similarities between *L. trifolii* and *O. biroi* were obviously higher than that between *L. sativae* and *O. biroi* (0.3936 and 0.0835, respectively). The temporal similarities of them were 0.4238 and 0.1112, respectively, while the spatial similarities of them were 0.9287 and 0.7512, respectively (Tables 1–3).

### Competition analysis among *L. sativae*, *L. trifolii* and *O. biroi*

The spatiotemporal competition coefficient between *L. sativae* and *L. trifolii* was 0.5871, which was obviously higher than the coefficients for intraspecific competition in *L. sativae* (0.3496) and in *L. trifolii* (0.3670) (Table 4).

**Table 4:** Competition coefficients of the two leafminer species, *Liriomyza sativae* (LS) and *L. trifolii* (LT) and the pupal parasitoid, *Opius biroi* (OB).

Competition coefficient	Temporal			Spatial			Spatiotemporal		
	LS	LT	OB	LS	LT	OB	LS	LT	OB
LS	0.4491			0.7783			0.3496		
LT	0.7085	0.3804		0.8287	0.9648		0.5871	0.3670	
OB	0.4870	0.1071	0.3435	0.9872	0.8418	0.7762	0.4808	0.0901	0.2667

The spatiotemporal competition coefficient between *L. sativae* and *O. biroi* (0.4808) was higher than that between *L. trifolii* and *O. biroi* (0.0901). This was also true for the spatial competition coefficient (0.9872 and 0.8418, respectively) and the temporal competition coefficient (0.4870 and 0.1071, respectively) (Table 4).

## Discussion

The importance of the ecological niche and competition in modern ecology and population biology is reflected in the fact that niche theory and competition theory have often been closely associated^27, 28^. *L. trifolii* and *L. sativae* are two closely related pest leafminers, when found occurring sympatrically, often exhibit serious competitive interactions^14^. In the present study, a wider spatiotemporal niche breadth and lower parasitism rate were found in *L. trifolii* than in *L. sativae*.

Interspecific competition is an almost unavoidable consequence of niche overlap^27^. The niche overlap between *L. sativae* and *L. trifolii* on our experimental bean plants was ca. 0.6, which showed that relatively serious resource competition was occurring between the two leafminer species. Niche breadth is the most important metric in reflecting inter- and intraspecific interactions^29^. Based on our analysis, a wider spatiotemporal niche was found in *L. trifolii* than in *L. sativae* when reared on kidney beans, indicating that the former leafminer species had an advantage over the latter in exploiting the limited available resources. The wider spatial niche of *L. trifolii* indicated that this species had a greater ability to occupy spatial cells, while the larger temporal niche of *L. sativae* showed that it occurred more frequently during plant growth. In addition, a wider niche breadth of *L. trifolii* (0.5002) than *L. sativae* (0.3879) on field bean plants was also found in Sanya, Hainan, during 2012–2013, which provides support for this study in terms of evidences from the field (unpublished).

Natural enemies play an important role in interspecific interactions by influencing competitive interactions through changes in the population densities of competing species^7^. We found that *O. biroi* had higher niche overlap with the leafminer *L. sativae* than its closely related species, *L. trifolii*, when they were both reared on kidney bean, showing that *O. biroi* preferred to seek out and parasitize *L. sativae* larvae more than those of *L. trifolii*. If asymmetric effects occur in both of the competitors, this could indirectly affect the direction of competition. Therefore, the asymmetric parasitoid effect found in the present study should favour *L. trifolii* when the three species occurring sympatrically.

The coefficients of interspecific competition between *L. sativae* and *L. trifolii* were higher than those for intraspecific competition, which could potentially drive competitive interaction between the two species. The relative strengths of interspecific and intraspecific competition can determine the direction of species coexistence or exclusion^30^. Our results indicating higher interspecific competition and higher control effects of the pupal parasitoid on *L. sativae* provide alternative cues for competitive exclusion occurring in the United States, Japan and China following the invasion of *L. trifolii*, although different exclusion directions were noted.

*L. trifolii* gradually displaced the dominant pest *L. sativae* in Hainan, China, from 2005–2013^31^. Moreover, a similar displacement process is proceeding in its area of invasion, such as in south Jiangsu, China^32^. Most papers published addressing the factors that mediate competitive displacement between *L. trifolii* and *L. sativae* have focused on the effects of environmental differences or human-mediated influences, including insecticides^15, 17^, cropping systems^18^, temperature variations^33^ and parasitoids^25^. Beyond these environmental factors, the inherent mechanisms of this displacement are still unclear. In this study, we explored the competitive interactions between two closely related species based on niche data; the results will help to enrich the theory of species displacement and can ultimately provide timely information that is useful for the management of future biological invasions.

## Materials and Methods

### Ethics statement

Our study involved populations of *L. trifolii*, *L. sativae* and *O. biroi*, which are not endangered or protected. The experimental locations are not privately owned or protected. No ethical approval or specific permissions were required for this study.

### Study site

This study was conducted in a vegetable field at the Sanya Research Institute of Agricultural Sciences (18.25° N, 109.53° E), Sanya, Hainan province.

### Insect preparation

Populations of *L. sativae* and *L. trifolii* were collected from field cowpeas, *Vigna unguiculata* (L.), in Sanya, Hainan Province, China in 2012. They were subsequently cultured on kidney bean, *Phaseolus vulgaris* L., at 26 ± 1 °C, humidity: 75% with a 14:10 light: dark photoperiod. The two species were reared for more than 3 generations to ensure the homogeneity of each population before being used in the experiments.

Populations of *O. biroi*, which are naturally occurring, entered the experimental field through the sieve opening of the cage. The parasitoid adults were ca. 0.18 mm in body width^26^ (while that of leafminer flies is greater than 0.5 mm), and the mesh size of the screen was 0.25 mm (measured by ourselves).

### Field cage setting

The field cage, which was made of nylon mesh, was 4 m long, 4.5 m wide and 2.5 m high (typical vegetable garden size among Chinese farmers). Field cage experiments were conducted from May to August, 2013 in Sanya, Hainan province. On 6 May, we planted the most common host plant of *Liriomyza* spp. (kidney beans, var. ShuangQing No. 1 - the widely adopted kidney bean variety that is most often planted in Hainan province) at a density of 20 plants per m^2^ inside the cage. We released 400 recently emerged adults of *L. trifolii* and 400 *L. sativae* into the cage after the plants had grown to ca. 40 cm in height.

### Surveying *Liriomyza* spp. and their parasitoid

We began to survey the populations of *Liriomyza* spp. and their parasitoids on 24 May. After ten healthy and uniform plants were selected for the survey (there were 5 replicates, and 2 plants within each replicate), the vertical space of each plant was divided into five grid cells: 0~30 cm, 30~60 cm, 60~90 cm, 90~120 cm and higher than 120 cm above the ground. We sampled 3 leaves in each cell for each survey (a total of 150 leaves were collected). The excised leaves were taken into the incubator, where identifications were conducted of the *Liriomyza* leafminer species and their parasitoids after their eclosion. The plants were surveyed every 4 days until the beans were harvested. No pesticides were used at any time during the survey period.

### Calculation of niche parameters and data analysis

**Niche breadth** was calculated according to Levins (1968)^34^:

1 $$B=1/(S\sum _{i=1}^{S}{P}_{i}^{2})$$

where *S* represents the resource states (i.e., spatial or temporal cells are available in this study), and *P*_*i*_ is the proportion of individuals of a species that is associated with *S*.

**Niche overlap** was also calculated from Levins (1968)^34^:

2 $${L}_{ij}=\sum _{h=1}^{S}{P}_{ih}{P}_{jh}({B}_{i})$$

where *P*_*ih*_ and *P*_*jh*_ are the proportions of the individuals of species *i* and *j*, respectively, that are associated with resource state *h* (i.e., one of the spatial or temporal cells in this study), and *B*_*i*_ is the niche breadth of species *i*.

**Niche proportional similarity** was calculated according to Colwell & Futuyma (1971)^35^:

3 $${C}_{ij}=1-1/2\sum _{h=1}^{S}|{P}_{ih}-{P}_{jh}|$$

where *P*_*ih*_ and *P*_*jh*_ are the same as above.

**The competition coefficient** was calculated according to May (1975)^36^:

4 $$A=\sum {P}_{i}{P}_{j}/[(\sum {P}_{i}^{2})(\sum {P}_{j}^{2})]$$

where *P*_*i*_ is the same as in eq. (1), and *P*_*j*_ is the proportion of individuals of species *j* that are associated with the resources state.

**The two-dimensional niche** was calculated according to Cody (1974)^37^ and May (1975)^36^:

5 $${B}_{TS}={B}_{T}\times {B}_{S}$$

where *B*_*T*_ and *B*_*S*_ are the temporal and spatial niches of the species, respectively.

All the niche parameters were analysed based on the population dynamics of the whole surveyed field^38–40^. Data analyses were conducted using IBM SPSS Statistics 19.0 (SPSS Inc., Chicago, IL, USA). We used one-way analysis of variances (ANOVAs) with Tukey’s HSD test to analyse the data. All percentage data were arcsine square root transformed before being analysed.
